# The Potential of the MAGIC TOM Parental Accessions to Explore the Genetic Variability in Tomato Acclimation to Repeated Cycles of Water Deficit and Recovery

**DOI:** 10.3389/fpls.2015.01172

**Published:** 2016-01-05

**Authors:** Julie Ripoll, Laurent Urban, Nadia Bertin

**Affiliations:** ^1^UR1115 Plantes et Systèmes de cultures Horticoles, INRAAvignon, France; ^2^Laboratoire de Physiologie des Fruits et Légumes EA4279, Université d’Avignon et des Pays du VaucluseAvignon, France

**Keywords:** fruit quality, MAGIC population, recovery period, *S. lycopersicum* L., water deficit

## Abstract

Episodes of water deficit (WD) during the crop cycle of tomato may negatively impact plant growth and fruit yield, but they may also improve fruit quality. Moreover, a moderate WD may induce a plant “memory effect” which is known to stimulate plant acclimation and defenses for upcoming stress episodes. The objective of this study was to analyze the positive and negative impacts of repeated episodes of WD at the plant and fruit levels. Three episodes of WD (–38, –45, and –55% of water supply) followed by three periods of recovery (“WD treatments”), were applied to the eight parents of the Multi-Parent Advanced Generation Inter-Cross population which offers the largest allelic variability observed in tomato. Predawn and midday water potentials, chlorophyll *a* fluorescence, growth and fruit quality traits [contents in sugars, acids, carotenoids, and ascorbic acid (AsA)] were measured throughout the experiment. Important genotypic variations were observed both at the plant and fruit levels and variations in fruit and leaf traits were found not to be correlated. Overall, the WD treatments were at the origin of important osmotic regulations, reduction of leaf growth, acclimation of photosynthetic functioning, notably through an increase in the chlorophyll content and in the quantum yield of the electron transport flux until PSI acceptors (*J*_0_^RE1^/*J*^ABS^). The effects on fruit sugar, acid, carotenoid and AsA contents on a dry matter basis ranged from negative to positive to nil depending on genotypes and stress intensity. Three small fruit size accessions were richer in AsA on a fresh matter basis, due to concentration effects. So, fruit quality was improved under WD mainly through concentration effects. On the whole, two accessions, LA1420 and Criollo appeared as interesting genetic resources, cumulating adaptive traits both at the leaf and fruit levels. Our observations show that the complexity involved in plant responses, when considering a broad range of physiological traits and the variability of genotypic effects, represent a true challenge for upcoming studies aiming at taking advantage of, not just dealing with WD.

## Introduction

Drought is a major threat for crop yield and improving agricultural productivity while reducing water use is a major issue. Indeed drought events are expected to increase in intensity, frequency, and geographic extent as a consequence of global change. Maintenance of plant productivity under limited water supply is a stress tolerance/acclimation trait, which shows inter- and intra-specific variation. In a recent review on fleshy fruits, Ripoll et al. (2014) championed the idea that drought can have positive effects on fruit quality while yield reduction could be minimized. However, developing plants adapted to drier conditions requires a better understanding of the physiological responses to WD. Many mechanisms may be involved in yield maintenance under WD conditions; in particular those involved in the reduction of water losses, in resource acquisition and allocation between source and sink organs, and in protection against oxidative stress (Ripoll et al., 2014). Exploring the existing genetic diversity in such traits opens new perspectives to address current challenges in the context of climate change. In tomato, current breeding methods have intensively selected yield or quality traits, while less attention has been paid to tolerance traits to abiotic stresses (Causse et al., 2001; Saliba-Colombani et al., 2001). MAGIC populations are an interesting source of genetic variability, since they display the largest allelic variability observed in different populations (Cavanagh et al., 2008). The eight parents of the MAGIC TOM *S. lycopersicum* L. have been selected for their higher rate of SNP differences among 360 others tomato accessions (Ranc, 2010), but to our knowledge these accessions were poorly characterized under stress conditions (**Table 1**). RIL populations have been developed from the MAGIC TOM population; they are used for genetic studies and breeding programs (Pascual et al., 2015). For instance a RIL population derived from an intraspecific cross between Cervil and Levovil, two of the eight MAGIC TOM parents (Saliba-Colombani et al., 2001), was used to identify QTLs of fruit quality (Chaïb et al., 2006). More recently, 119 recombinant inbred accessions derived from the same cross have been phenotyped and genotyped under two water regimes (control and moderate WD). This study revealed 11 interactive QTLs which determine genotypic expression as a function of watering regimes and which are associated to plant and fruit quality traits (Albert et al., 2015). This study concluded that large fruit tomatoes are more sensitive to drought than cherry tomatoes and that breeding for crop performance under conditions of deficit irrigation should aim at achieving trade-offs between fruit quality and yield.

**Table 1:** Some characteristics of the eight parents of the MAGIC TOM population selected for their high degree of allelic variability (Ranc, 2010).

Genotype	Cultivar	Fruit weight (g)	Duration of cell division (days)	Duration of cell expansion (days)	Duration of ripening (days)	Known resistance to stressor
Cervil	*S. lycopersicum* ‘cerasiforme’	<5	14	25	10	Sensitive to saline stress (Manaa et al., 2011)
Criollo	*S. lycopersicum* ‘cerasiforme’	<15	21	24	10	No reference
LA1420	*S. lycopersicum* ‘cerasiforme’	<50	21	24	10	No reference
Plovdiv XXIVa	*S. lycopersicum* ‘cerasiforme’	<50	20	25	10	No reference
Stupicke Polni Rane	*S. lycopersicum* ‘esculentum’	<70	21	24	10	1. Stomatal closure after five days without irrigation (Hnilickova and Duffek, 2004)
						2. Resistant to *Phytophthora infestans* (Petrikova et al., 2003)
						3. Increased photosynthesis after 4 days at cold temperature (Hnilickova et al., 2002)
						5. Strong emission of volatiles (Subrtova et al., 1985)
Ferum	*S. lycopersicum* ‘esculentum’	<130	25	26	10	No reference
LA0147	*S. lycopersicum* ‘esculentum’	<130	25	25	10	No reference
Levovil	*S. lycopersicum* ‘esculentum’	<130	25	25	10	Tolerant to saline stress (Manaa et al., 2011)

*All of them have an indeterminate growth pattern.*

Water deficit is known to impact the leaf physiological activity, usually resulting in a reduction of stomatal conductance, conducing to a reduction of photosynthetic activity, a decrease in growth and an increased risk of photo-oxidative stress (Tardieu et al., 2006). However, during RPs, mechanisms of plant defenses or acclimation are expected to be exacerbated by WD thanks to priming mechanisms (Bruce et al., 2007). Moreover, two successive stress periods may stimulate water uptake during the second stress period, resulting in a reduction of the negative impact of WD on plant growth (Al Gehani, 2005). During RP, growth may not completely recover depending on the duration and the intensity of WD. In tomato, when water supply is suppressed during the reproductive period (from 9 to 13 days), leaf water potential, stomatal conductance, and net photosynthesis rate can recover their initial values (Rahman et al., 1999). Cell wall extensibility which plays an important role in cell expansion, is less likely to recover after drought stress, arguably due to the rapid accumulation of abscisic acid (Mahdid et al., 2011). However, in tomato, it has been observed that some Mediterranean drought adapted landraces tend to have thinner, more elastic cell walls, which allow them to maintain cell turgor by reducing cell volume, when cultivated under drought (Galmés et al., 2011). Under extreme WD, recovery may be partial due to damage on PSII. Indeed the synthesis of reactive oxygen species can increase under WD, and recovery depends on the quantity produced vs. the quantity scavenged (Xu et al., 2010). More generally, it has been demonstrated that “plant memory” of stress induces a faster activation of response mechanisms to other stressors (abiotic or biotic stress) through the common hormonal response pathways (Li and Zhang, 2012). The faster activation of defense response in primed plants is associated to an increased gene expression and to the accumulation of inactive signaling proteins and transcription factors (Bruce et al., 2007).

Regarding fruit quality (e.g., contents in soluble sugars, organic acids, carotenoids or C vitamin), the impact of WD may differ according to the stage of fruit development at the time of WD (reviewed in Ripoll et al., 2014). When WD occurs during the cell division stage, it may induce carbon starvation that negatively regulates cell division and consequently final fruit size. However, a positive effect on carbon supply to the remaining fruits has been suggested due to a negative regulation of fruit setting and fruit load. In peach (*Prunus persica* L.), WD has been shown to improve fruit sweetness, flavor and fruit size when applied during the stage of cell division and rapid endocarp hardening (Li et al., 1989; Vallverdu et al., 2012). In tomato, a moderate WD applied during the stage of cell division does not reduce fruit size, arguably due to important osmotic regulations (Ripoll et al., 2015). During the cell expansion stage, WD mainly impacts water flows between source and sink organs (Münch, 1930) through osmotic and turgor regulations. In peach, negative effects on yield have been observed associated with a decrease in fruit water content (Li et al., 1989; Girona et al., 2004). During ripening, which coincides with seed maturation, progressive softening, accumulation of pigments, sugars, and acids, and release of volatiles (Osorio et al., 2013), WD may interact with ethylene synthesis (Fray et al., 1994; Barry and Giovannoni, 2007). In tomato, moderate WD during ripening reduces the accumulation of some carotenoids, whereas the effects on sugar accumulation seem to be genotype dependent (Ripoll et al., 2015). Different combinations of WD applied during flowering and fruit growth, or during flowering and ripening, showed that the improvement of fruit quality is counterbalanced by the decrease in yield when at least one development phase is exposed to intensive stress in oranges *Citrus sinensis* L. (García-Tejero et al., 2010). Thus, WD episodes followed by RP may impact fruit quality in a different way from single WD episode. For instance, fruit carotenoid content increases in tomato plants grown under WD and this increase is exacerbated after a second period of WD due to an increase in antioxidant enzyme activity during both the first WD period and the RP (Stoeva et al., 2010, 2012). So understanding the effect of WD on fruit quality is a complex issue due to the numerous factors involved, even though WD is generally expected to improve fruit quality (Ripoll et al., 2014). Similar observations have been made in response to moderate salt stress, involving similar mechanisms (e.g., Munns, 2002; De Pascale et al., 2007). Finally it appears that one could take advantage of the “memory effect” induced after a moderate WD, in order to promote fruit quality while minimizing yield reduction. Even though there is some evidence that WD can be used as a lever to increase quality of fruits, there is a lack of references about the effect of WD episodes of increasing intensity followed by periods of recovery.

In the present study, our objectives were: (i) to provide an overview of the beneficial and detrimental impacts of WD treatments at the plant and fruit levels, (ii) to assess the genetic variability of these responses, and (iii) to reveal interesting traits of plant acclimation to WD, which could be used in future breeding programs. The work was performed on the eight parents of the MAGIC TOM population. Plant and fruit responses were measured during three successive periods of WD of increasing intensity followed by RP (“WD treatments”), which is clearly an original feature of the present study. Moderate WD was achieved by reducing the supply of water by about 38% during the first WD period, 45% during the second WD period and 55% during the last WD period when compared to control plants. Predawn water potential, stem water potential at midday, chlorophyll content and chlorophyll *a* fluorescence as well as leaf composition (soluble sugars and organic acids) were assessed before and after each WD period. Fruit quality (soluble sugars, organic acids, carotenoids, and AsA contents) was measured on two batches of fruits which developed at different periods of the WD treatments.

## Materials and Methods

### Plant Material and Experimental Conditions

The eight parents of the MAGIC TOM (**Table 1**) encompass four large-fruit accessions [Ferum, LA0147, Levovil, and Stupicke Polni Rane (here called Stupicke)], and four cherry accessions [Cervil, Criollo, LA1420, and PlovdivXXIVa (here called Plovdiv)]. All are indeterminate tomatoes. LA1420 seeds were provided by the Tomato Genetics Resource Centre, Davis, CA, USA. Cervil and Levovil seeds were provided by Vilmorin Seed Company. The other accessions were supplied by the Tomato Genetic Resource Centre of INRA, Avignon, France (Causse et al., 2013).

**FIGURE 1:**
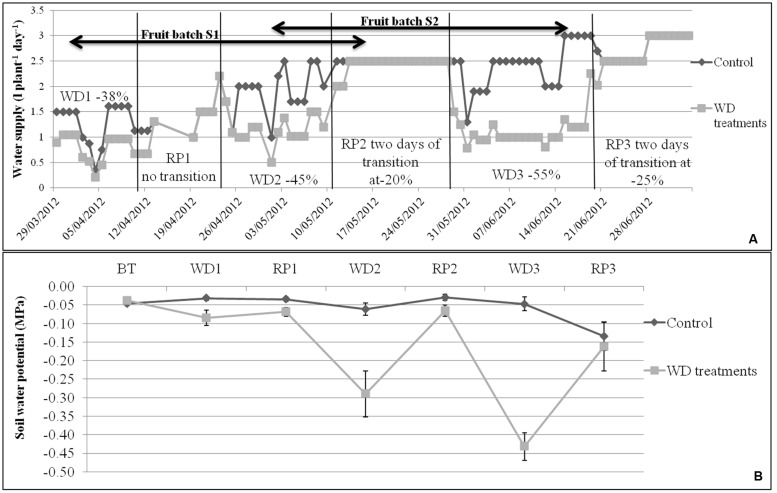
**(A)** Water supply (water in l plant^-1^ day^-1^) for control and WD treatments, and **(B)** mean soil water potentials during each WD and RP period (*n* = 8 ± SE), during the experiment period. Irrigation of control plants was monitored according to the measured potential evapotranspiration. The WD treatments consist in 3 cycles of WD of increasing intensity (–38, –45, and –55%) followed by RP periods. Transition periods of 2 days were applied after each WD period in order to reduce the risk of fruit blossom end rot. The fruit development periods of S1 and S2 lots are indicated by arrows.

The experiment was conducted during spring and summer 2012 in a glasshouse located near Avignon, France. Irrigation was calculated according to daily ETP (Penman, 1948), taking into account crop coefficients (*K*c = 40% before treatments, 50–75% during the first WD, 75–100% from the first RP and 100% until the end of the experiment). The control irrigation met the evaporative demand. The WD treatments corresponded to three phases of WD of increasing intensity (–38, –45, and –55% of water supply when compared to control plants) followed by three RP (**Figure 1A**). Each WD and RP period lasted approximately 15 days. During recovery, WD was first reduced by half for 2 days and then brought back to the control level. Climate conditions (temperature, humidity, and light intensity) in the glasshouse were recorded every minute and data were averaged hourly throughout the experiment. Average day and night temperatures were stable until 12 June, i.e., until RP2 (around 25°C and 18°C, respectively). Temperatures increased during WD3 and RP3 (around 30°C and 22°C, respectively) due to seasonal effects. At the same time, the daily light integral increased in the glasshouse (from 5 to 11 MJ m^-2^ day^-1^), whereas average day and night humidity decreased (from 57 to 37% at daytime and from 80 to 60% at night) from WD1 to RP3. Plants were grown in 4 L pots filled with compost (substrate 460, Klasmann, Champety, France) distributed in two rows (control and stressed plants) of 80 plants each (10 plants per genotype) surrounded by border plants. The density was 1.3 plant m^-2^.

The soil water potential (Ψ_soil_) was measured daily with Watermark probes (Watermark 253, Campbell Scientific, Antony, France) placed at the opposite of the drippers (**Figure 1B**). One probe per treatment per genotype was used and connected to a data logging system (Data logger, Campbell Scientific, Antony, France). Results were converted into MPa using equation 8 given by Shock et al. (1998). In control conditions, Ψ_soil_ was rather stable until the WD3 period (-0.04 ± 0.01 MPa) and decreased during RP3 due to plant development (–0.05 ± 0.01 MPa). On the contrary, the soil water potential decreased to –0.09 ± 0.02, –0.29 ± 0.06, and –0.43 ± 0.04 MPa during WD1, WD2, and WD3, respectively. Nutrients were applied daily using a commercial solution (Liquoplant Rose, Plantin, Courthézon, France).

Flowers were pollinated three times a week using an electrical bee. Trusses were pruned according to final fruit weight (**Table 1**) in order to obtain comparable levels of competition among fruits for all genotypes (Cervil: 12 fruits per truss, Criollo: 10 fruits, LA1420: eight fruits, Plovdiv: eight fruits, Stupicke: six fruits, Ferum: five fruits, LA0147: five fruits, Levovil: four fruits). No chemical treatment was applied and *Macrolophus caliginosus* were released throughout the culture to protect plants from whiteflies.

### Plant Measurements

Stem water potential was measured using a pressure chamber (SAM Précis 2000 Gradignan, France) at predawn and at solar noon (Ψ_predawn_ and Ψ_midday_) at the end of each WD and RP period (*n* ≥ 4 for a given genotype, 64 plants minimum). Leaves were bagged the day before, at nightfall.

Fluorescence of chlorophyll *a* was measured on dark adapted leaves (30 min.) using a fluorimeter (HANDY-PEA, Hansatech, King’s Lynn, UK). Dark-adaptation allowed the PSII electron acceptor pool to be gradually re-oxidized to a point where all PSII reaction centers are capable of undertaking photochemistry. Measurements were carried out with an induction period of 1 s and leaves were illuminated to a light level of 3000 μmol photons m^-2^ s^-1^. The measurements were carried out on non-senescent mature leaves, at around 11 a.m. during the last three days of each period (*n* ≥ 4 for a given genotype, 64 plants minimum). The maximum photochemical efficiency of light harvesting of PSII (*F*_V_/*F*_M_), the PI of Strasser et al. (2004) and the quantum yield of the electron transport flux until PSI acceptors (*J*_0_^RE1^/*J*^ABS^; Stirbet and Govindjee, 2011) were calculated. The chlorophyll content was evaluated using a chlorophyll meter (SPAD 502, Konica–Minolta, Osaka, Japan) on adjacent leaves.

Plant leaf number and leaf length were measured at the end of each period (*n* ≥ 5). The last day of the WD3 period, two non-senescent mature leaves, which were initiated during the WD treatments, were harvested on each plant and their specific leaf area (*n* ≥ 5, 80 plants min.) was measured. Leaf area was measured with a Planimeter (Li-3100 C Area Meter, Li-Cor, Lincoln, NE, USA) and leaf dry weight was measured after seven days at 70° C in a ventilated oven.

Furthermore, at the end of each WD and RP period four leaflets of two mature leaves were harvested around 11 a.m. on five plants per genotype per treatment (80 plants in total), immediately frozen in liquid nitrogen and stored at –80°C, for biochemical analysis.

### Fruit Measurements

The dates of anthesis of the successive trusses were recorded on all plants during the whole experiment. Thus, fruits could be pooled according to the developmental stage at the time of the WD treatments. The first pool of fruits (S1) was harvested during RP2, whereas WD1 occurred during the cell division period and WD2 during the cell expansion period. For the second pool of fruits (S2), WD2 occurred during the cell division period and WD3 during the cell expansion and ripening periods (**Figure 1A**). Fruit setting and abortion were recorded on the first eight trusses of each plant.

All measurements were performed on red mature fruits (breaker stage plus at least five days) harvested on five plants per genotype and per treatment (80 plants in total). Fruits were harvested at 11 a.m., avoiding the first proximal and the last distal fruits of each truss. Fruit diameter and fresh weight of all fruits were measured. Then fruits were frozen in liquid nitrogen and kept at –80°C prior to biochemical analysis of pericarp soluble sugars, organic acids, carotenoids, AsA, starch (only for Cervil), and DM contents. For biochemical analyses, fruits were pooled into five batches of three to five fruits for each treatment and genotype.

### Biochemical Analyses

Soluble sugars and organic acids were extracted according to Gomez et al. (2002) and measured by HPLC method. Starch was measured on the supernatant after hydrolysis. The glucose released by starch hydrolysis was measured using the micro-method of Gomez et al. (2007) and starch content was calculated. DM content was measured after lyophilisation.

Assays of total, reduced and oxidized AsA content were carried out on ground powder conserved at –80°C using microplates and a plate reader, as previously described by Stevens et al. (2006). The absorbance was read at 550 nm. The specificity of the assay was checked by comparison with other known methods (Stevens et al., 2006). Carotenoids were extracted according to the method of Serino et al. (2009) and assayed by HPLC.

### Statistical Analyses

All statistical analyses were performed using R3.1.0 (R Core Team, 2014). The evolution of physiological parameters over the experiment was compared between stressed and control plants using the AUCs. AUCs were calculated according to the Trapezoidal rule (Atkinson, 2008). Genotype and treatment effects on all parameters were analyzed by two-way ANOVA. The residue normality (ANOVA) was tested using the Shapiro–Wilk test (Royston, 1995). Levene’s test was used to verify homoscedasticity of variances of the residues (Car package; Fox and Weisberg, 2011). When authorized, two-way ANOVA was performed and followed by multiple comparisons of means (Tukey test, lsmeans and multcompView packages; Graves et al., 2012 and Lenth, 2014, respectively). Alternatively, we used the non-parametric Kruskal–Wallis test (pgirmess package; Giraudoux, 2014). Heat-maps of the fruit traits were plotted according to control and WD treatments (gplots package; Warnes et al., 2014). Partial correlations network was built on plant and fruit data, based on the residues of the linear regressions (elimination of the genotype effect) and performed independently for the control and for the WD treatments (*P* threshold < 0.001; GGMselect, GeneNet, and igraph packages; Giraud et al., 2009; Schaefer et al., 2014, and Csardi and Nepusz, 2006, respectively). Finally, clustering analysis was performed on leaf and fruit data (FactoMineR package; Husson et al., 2014).

## Results

### Mean Effects of the WD Treatments at the Plant and Leaf Levels

In order to evaluate the global plant response to the WD treatments, AUCs were calculated for Ψ_predawn_, Ψ_midday_, DM content, soluble sugars, organic acids, starch content, chlorophyll content, the maximum efficiency at which light absorbed by PSII is used for reduction of *Q*_A_ (*F*_V_/*F*_M_), the quantum yield of the electron transport flux until PSI acceptors *J*_0_^RE1^/*J*^ABS^ and the PI index (**Table 2**). AUCs represent the cumulated response from the onset of the WD treatments until the end of WD3 (**Figure 1**). RP3 was discarded because healthy non-senescent mature leaves were rare at this time.

**Table 2:** Relative differences in plant and leaf traits between the WD treatments and the control.

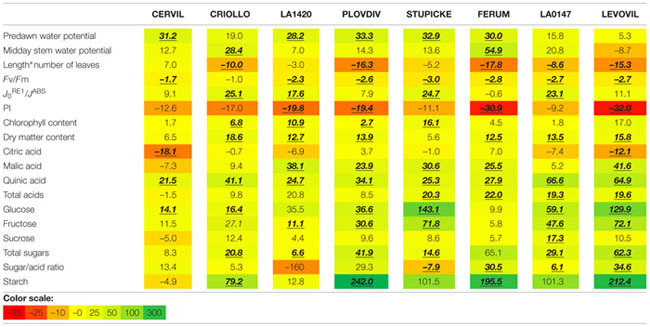

*Stem water potentials (in absolute values), leaf length × leaf number, leaf metabolite contents (soluble sugars, organic acids, and starch on a DM basis) and parameters related to leaf photosynthetic activity were measured at the end of the WD for the eight parents of the MAGIC TOM (ranked in order of increasing fruit size). Relative differences were calculated based on total AUCs as: Parameter (%) = 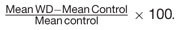
*

*The percentages were scaled by color (green for high and red for low values). Significant differences are indicated by bold, italic, and underlined fonts for P < 0.05 (Two-way ANOVA test or Kruskal–Wallis test).*

The *F*_V_/*F*_M_ index decreased in response to the WD treatments in all genotypes except in Criollo. AUCs of PI index also significantly decreased for four genotypes (–19.4% in Plovdiv, –19.8% in LA1420, –30.9% in Ferum, and –32% in Levovil). On the contrary, *J*_0_^RE1^/*J*^ABS^ increased in several genotypes (+25.1% in Criollo, +17.6% in LA1420, +24.7% in Stupicke, +23.1% LA0147) as well as the relative chlorophyll content (+6.8% in Criollo, +10.9% in LA1420, +2.7% in Plovdiv, and +16.1% in Stupicke). Then, leaf DM content (**Table 2**) increased due to the WD treatments (except in Cervil and Stupicke) as well as the contents in malic (except Cervil, Criollo and LA0147) and quinic acids, in glucose (except LA1420 and Ferum), in fructose (except Cervil, Criollo, and Ferum), and in starch (except Cervil and LA1420). The plant leaf area, assessed through leaf size and leaf number, significantly decreased in all genotypes except Cervil, LA1420, and Stupicke.

**FIGURE 2:**
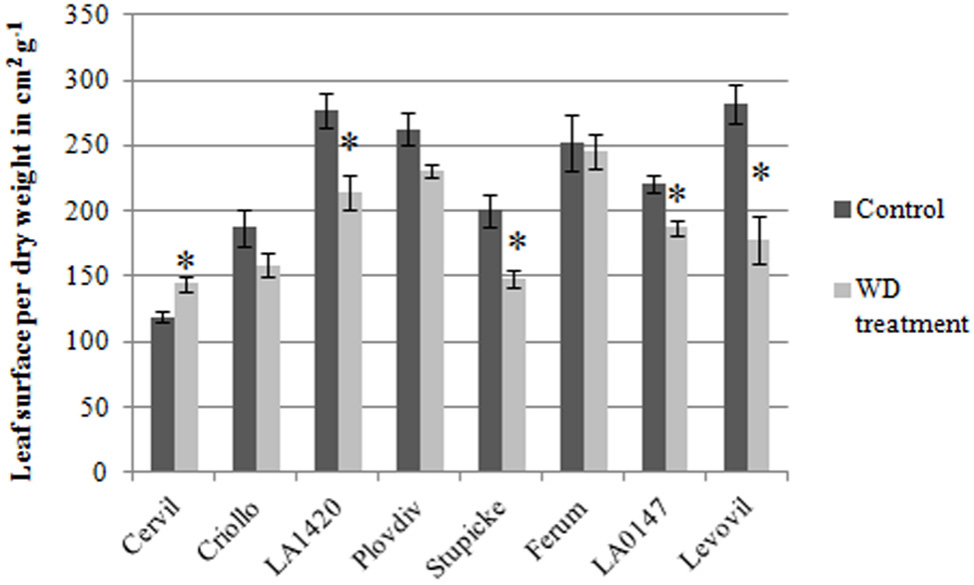
**Specific leaf area of the eight parents of the MAGIC TOM, measured at the end of the trial (control and WD treatments).** Data are means ± SE (*n* ≥ 5). Significant differences between control and WD treatments are indicated by stars for *P* < 0.05 (Student test performed for each genotype).

The specific leaf surface area measured at the end of the experiment on non-senescent mature leaves varied by a factor three among genotypes and it significantly decreased in response to the WD treatments in LA1420 (–26%), Stupicke (–26%), LA0147 (–15%) and Levovil (–36%) while it increased in Cervil (+21%), (**Figure 2**).

### Effects of the WD Treatments on Fruit Size and Composition

**FIGURE 3:**
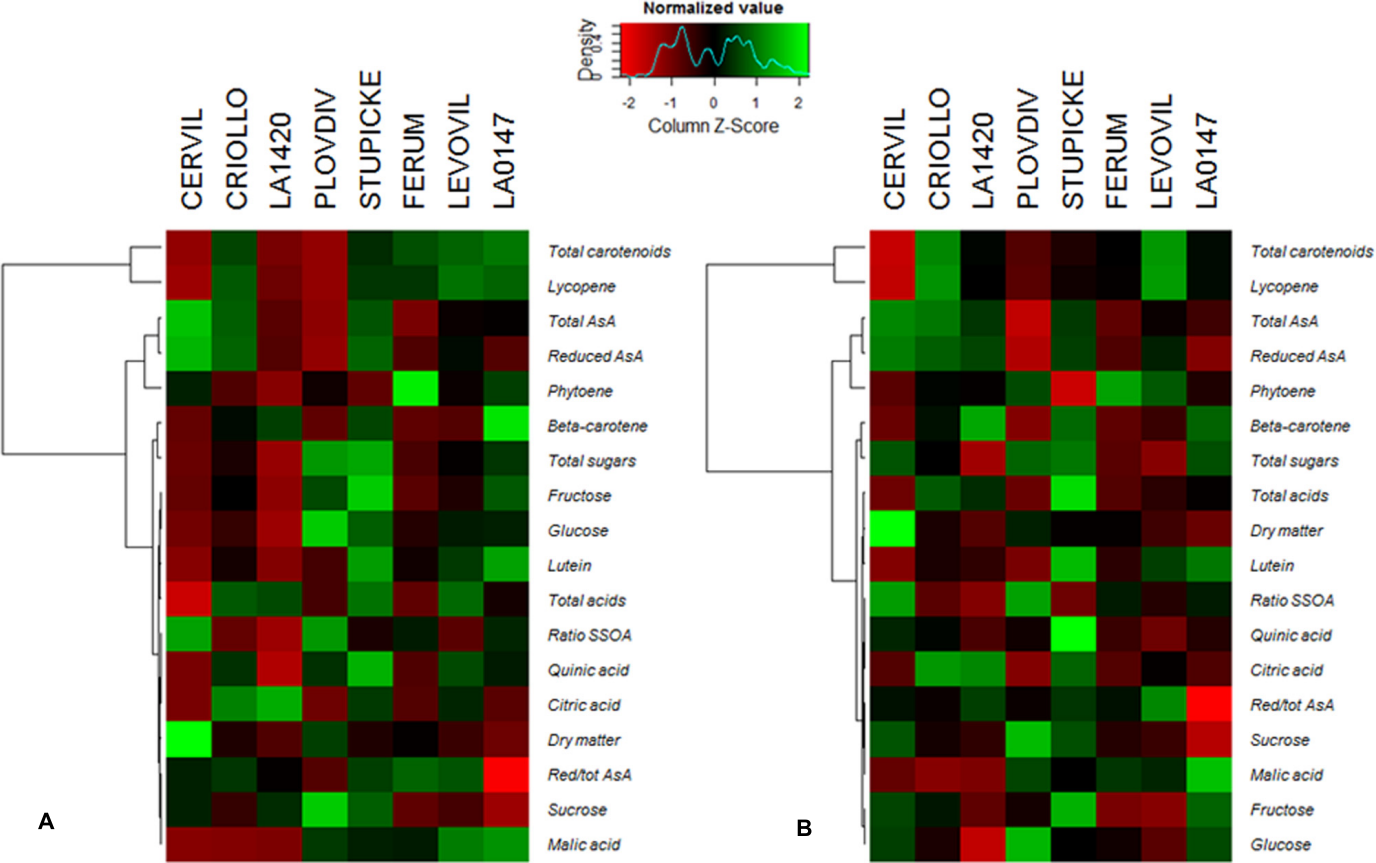
**Heat map representation of fruit composition (contents in soluble sugars, organic acids, AsA, and carotenoids expressed on a DM basis), for the parents of the MAGIC TOM population, grown under control conditions (A) and under the WD treatment (B) applied during the fruit cell division (WD1) and the cell expansion period (WD2; S1 fruits).** The WD treatments consisted in three WD periods of increasing intensity followed by RPs. Data are centered and scaled by parameters (*n* ≥ 5). Parameters are hierarchically classified according to Euclidean distances and ranged according to colors (green for high and red for low).

A hierarchical clustering analysis, based on fruit composition variations (on a DM basis) among genotypes, is presented in **Figure 3** for the first batch of fruits (S1). Clusters indicate fruit traits that co-varied under a given condition and highlight the differences in metabolite concentrations among genotypes. For both conditions, total soluble sugars, total organic acids, lutein, and β-carotene could be pooled together, as could be pooled together AsA and phytoene contents, on the one hand, and lycopene and total carotenoids contents, on the other hand. **Figure 3** highlights contrasted composition among genotypes in the control. For instance Cervil fruits which had the highest DM content, had the lowest content in total sugars, acids, carotenoids, and lycopene, but the highest content in total AsA on a DM basis. Similarly, LA1420 fruits were poor in all compounds except acids and β-carotene. On the contrary, Stupicke fruits were the richest in all compounds except phytoene. Similar results were observed in control fruits of the S2 fruits (data not shown).

Variations in fruit composition in response to the WD treatments are presented on a DM basis for S1 and S2 fruits (**Tables 3** and **4**, respectively). Though not significant, the decrease in fruit size and fresh mass was more pronounced in S2 fruits than in S1 fruits. For instance, on Levovil, the fruit fresh mass decreased by 42.8% in S2 fruits and by 13% in S1 fruits. Cervil S1 fruits were the less sensitive (**Table 3**). The number of set fruits measured on the eight first trusses was not significantly different between control and WD plants since inflorescences were pruned (data not shown).

**Table 3:** Relative differences in fruit metabolite contents between the WD treatments and the control.

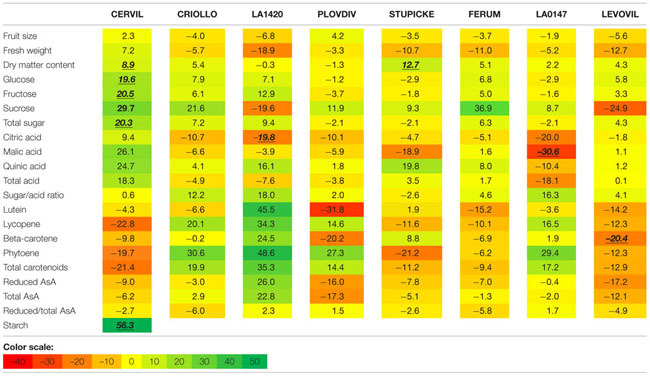

Soluble sugars, organic acids, AsA, and carotenoids were measured on a dry mass basis for the eight MAGIC TOM parents. Relative differences were calculated as described in the legend of **Table 2**. The percentages were scaled by color (green for high and red for low values). S1 fruits were harvested at RP2 after a first WD period during cell division (WD1) and a second WD period during cell expansion (WD2). Significant differences are indicated by red bold, italic, and underlined fonts for *P* < 0.05 (Two-way ANOVA test or Kruskal–Wallis test).

**Table 4:** Relative differences in fruit metabolite contents between the WD treatments and the control.

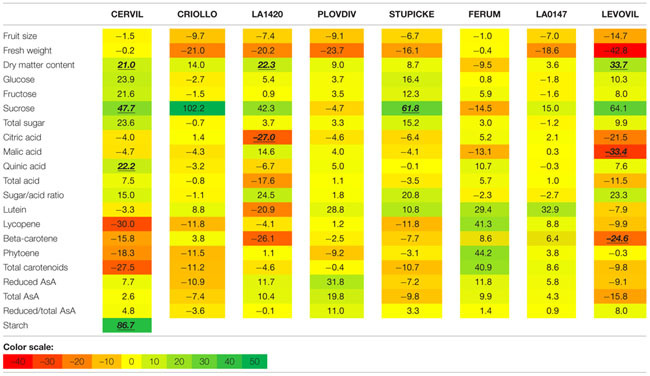

*Soluble sugars, organic acids, AsA, and carotenoids were measured on a dry mass basis for the eight MAGIC TOM parents. Relative differences were calculated as described in the legend of **Table 2**. The percentages were scaled by color (green for high and red for low values). S2 fruits were harvested at WD3 (Cervil, Criollo, Plovdiv, and Stupicke) and RP3 (Levovil, LA1420, LA0147, and Ferum) after the first WD period during cell division (WD2) and a second WD period during cell expansion and ripening (WD3). Significant differences are indicated by red bold, italic, and underlined fonts for P < 0.05 (Two-way ANOVA test or Kruskal–Wallis test).*

On a DM basis, the variations in fruit composition were significant for five genotypes (Cervil, LA1420, Stupicke, LA0147, and Levovil; **Table 3**, S1 fruits). In the case of Cervil fruit, an increase in DM content (+8.9%), total soluble sugars (+20.3%), and starch content (+56.3%) was observed. Acid contents dropped in LA1420 fruits (–19.8% citric acid) and in LA0147 fruits (–30.6% malic acid). DM content was higher in Stupicke fruits (+12.7%), without any change in DM composition. In Levovil fruits, the β-carotene content was significantly reduced (–20.4%). Interestingly the contents in lycopene and carotenoids increased in four genotypes (Criollo, LA1420, Plovdiv, and LA0147), whereas total AsA content decreased in all genotypes except LA1420. However, these variations were not significant. Consistent results were observed in S2 fruits (**Table 4**) except for LA0147 whose contents in lycopene, carotenoids and total AsA were hardly affected. The fruit DM content increased in all genotypes (except Ferum) and to a larger extend in Cervil (+21%), LA1420 (+22.3%), and Levovil (+33.7%). Among soluble sugars, the sucrose content was more affected than glucose or fructose contents (+47.7% in Cervil and +61.8% in Stupicke).

On a fresh matter basis, sugars and quinic acid contents were significantly higher under WD in Cervil (respectively, +31.3 and +48.5% total sugar content in S1 and S2 fruits, and, respectively, +36.3 and +46.9% quinic acid content in S1 and S2 fruits) and in Levovil (+47% of total sugar and +44% of quinic acid for the S2 fruits; data not shown). In Stupicke only the quinic acid content was higher under WD (+35.6%, S1 fruits). For the S2 fruits, reduced and total AsA contents were higher in Cervil, LA1420, and Plovdiv (respectively, +23.5, +31.2, and +30.3% reduced AsA, *P* < 0.05). So, metabolic and concentration effects added up for the compounds that increased both on a dry and fresh matter basis (mainly sugars and acids), whereas the negative effects of WD observed on a DM basis were mitigated by concentration effects, resulting in fruit quality homeostasis.

### Partial Correlation Network and Clustering Among Leaf and Fruit Traits for Control and WD Treatments

**FIGURE 4:**
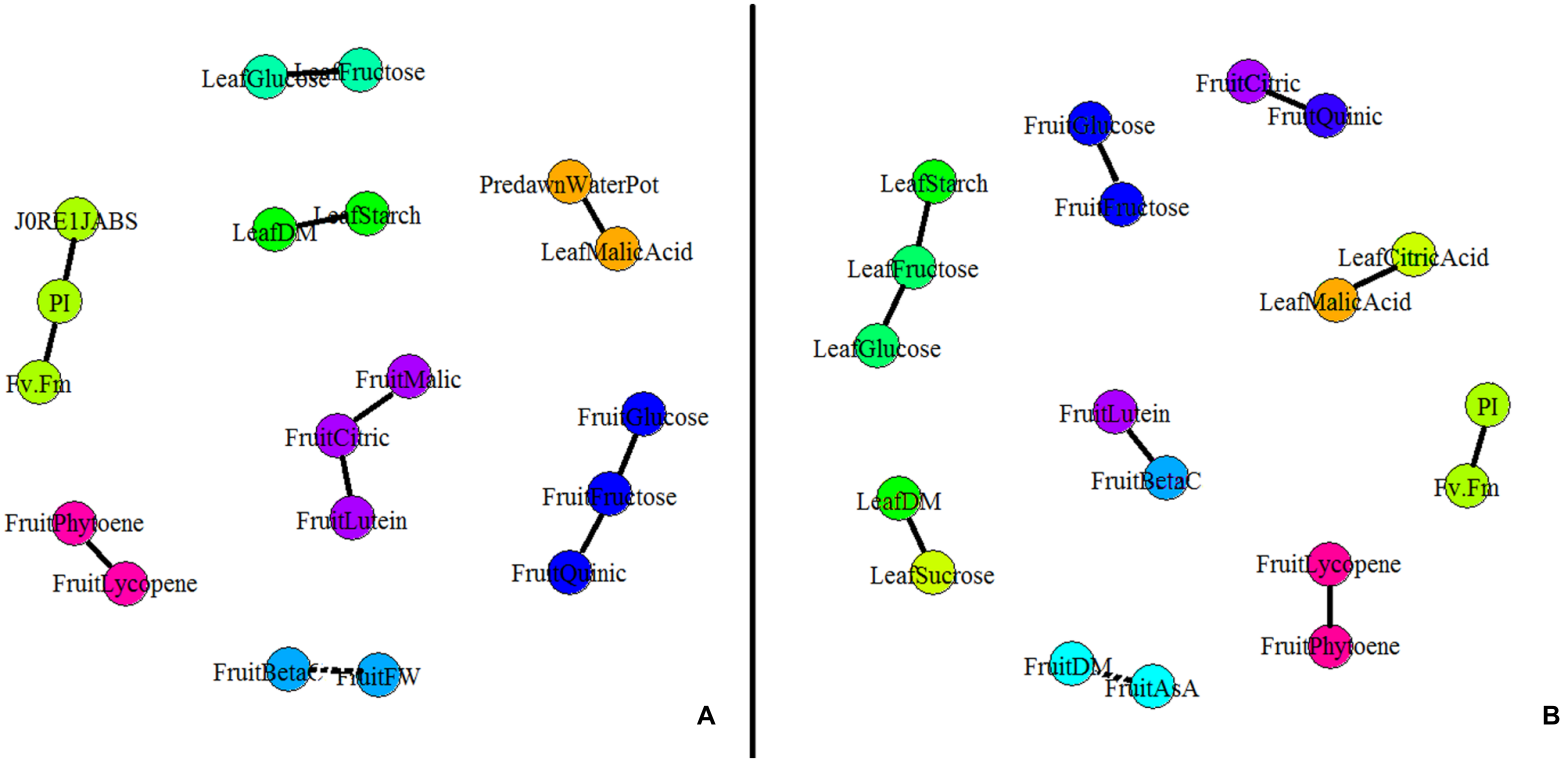
**Partial correlation network for plant and fruit composition [DM content, fruit fresh weight (FW), soluble sugars, organic acids, carotenoids, and AsA contents] and physiological parameters (the maximum quantum efficiency of PSII (*F*_V_/*F*_M_), the PI of Strasser and the quantum yield of the electron transport flow until PSI acceptors (*J*_0_^RE1^/*J*^ABS^).** Partial correlations were calculated on AUCs for plant measurements and for S2 fruits (*n* = 5, 80 plants) under **(A)** control and **(B)** WD conditions. The network was built using GGMselect, GeneNet, and igraph packages on R. Solid lines indicate positive correlations between parameters whereas dotted lines indicate negative correlations (*P* < 0.001).

A partial correlation network was built based on the AUCs calculated for the different leaf and fruit traits (**Figure 4**). Interestingly no leaf trait correlated to any fruit traits under both conditions. In control conditions, four independent leaf clusters emerged (**Figure 4A**). Positive correlations existed between leaf starch content and leaf DM content, between leaf malic acid content and Ψ_predawn_, and between leaf fructose and glucose contents. Then PI correlated with *J*_0_^RE1^/*J*^ABS^ and *F*_V_/*F*_M_. Concerning fruit traits, five independent clusters were found under control conditions. Fructose, glucose, and quinic acid contents were positively correlated one to each other, as well as lycopene and phytoene. Finally, fruit citric acid content, fruit malic acid content, and fruit lutein content were positively correlated one to each other while fruit fresh mass was negatively correlated with the fruit β-carotene content.

A different network was observed under the WD treatments when compared to the control (**Figure 4B**) suggesting that physiological acclimation processes were at play. At the leaf level, *J*_0_^RE1^/*J*^ABS^ did not correlate any more with PI and *F*_V_/*F*_M_ indexes. This observation suggests a regulation of the functioning of the photosynthetic machinery (**Table 2**). Leaf sugars (starch, fructose, and glucose) constituted an independent cluster. Leaf malic acid did not correlate anymore with Ψ_predawn_ but with leaf citric acid, due to the increase in organic acid contents (**Table 2**). Leaf DM content was positively correlated to leaf sucrose content instead of starch content. At the fruit level, the fruit DM content negatively correlated with the total ASA content, while the phytoene content was positively correlated with the lycopene content, as well as the lutein and β-carotene contents, the citric and quinic acid contents, and the glucose and fructose contents.

**FIGURE 5:**
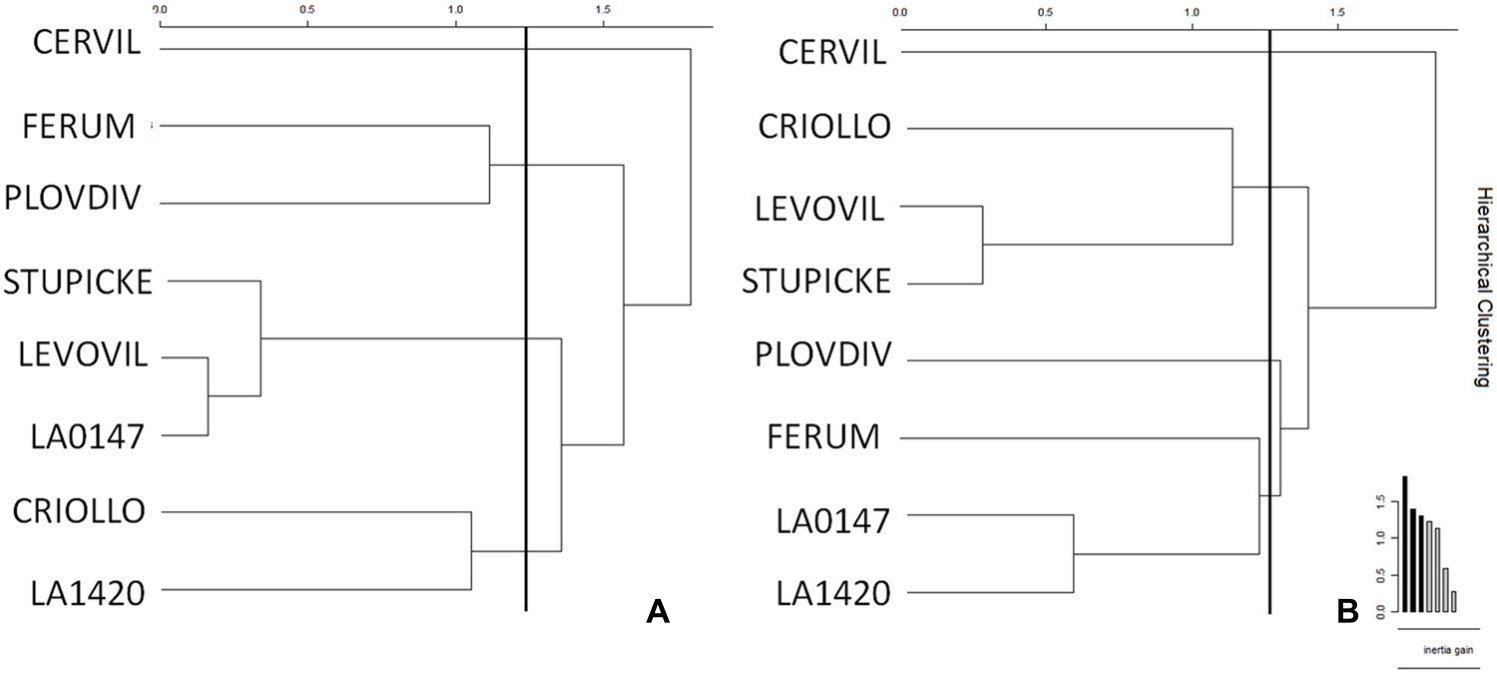
**Clustering of genotypes according to the AUCs for leaf and fruit traits for (A) control and (B) WD plants.** AUCs were calculated according to the trapezoidal rule for each variable measured at the end of each WD or RP (except RP3 which was not included in the area because of leaf senescence). AUCs are the integrals of the parameters over time computed for control and WD plants. The WD treatments consisted in three WD of increasing intensity followed by RPs. Clusters were realized depending on Euclidean distances (vertical bars in the graph) and inertia gains.

Clustering of the leaf and fruit traits measured in the experiment was realized for control (**Figure 5A**) and WD (**Figure 5B**) plants. Four clusters emerged for the control plants. LA1420 and Criollo were clustered according to their high fruit citric acid content and low leaf chlorophyll content. Levovil, Stupicke, and LA0147 stand out due to their similar malic acid and phytoene contents in fruits and by their low leaf glucose content. Ferum and Plovdiv were clustered due to their high PI index value and their low fruit AsA content. The cherry tomato Cervil constituted its own cluster due to its high starch, glucose, fructose, and malic acid contents in leaves and its high DM and low quinic acid contents in fruits. Similarly, four clusters emerged for the WD plants. Clustered genotypes did not necessarily respond to the WD treatments in the same way (**Table 2**). The first cluster includes Levovil, Stupicke, and Criollo, which have high fruit β-carotene content. LA1420, LA0147 and Ferum were clustered due to similar Euclidean distances without emergent traits. Plovdiv and Cervil constituted their own single cluster due to high glucose and low citric acid contents in leaves for Plovdiv, and to high DM content in fruits, high sucrose and fructose contents in leaves, and low Ψ_predawn_ and Ψ_midday_ values for Cervil.

## Discussion

### Leaf Responses to the WD Treatments Involved Osmotic and Photosynthetic Regulations

Current responses of plants submitted to WD encompass a decrease in plant water status and in water loss (as evidenced by a decrease in Ψ_midday_, in stomatal conductance and in transpiration rate), a reduction of leaf growth, and osmotic regulations (Tardieu et al., 2006). In the present study, osmotic adjustment and photosynthetic adaptation seem to have prevailed in the response to the WD treatments, as evidenced by the changes in leaf composition (increase in concentrations of soluble sugars and organic acids) and the relative stability of Ψ_midday_ except in Criollo and Ferum (**Table 2**). The absence of significant decrease in Ψ_midday_ may be explained by an increase in turgor. Indeed, when the elasticity modulus decreases, reflecting a decrease in cell wall rigidity, the decrease in turgor is mitigated during dehydration (Hsiao et al., 1976; Zimmermann, 1978), what probably happened in the present trial, although data are missing to substantiate this idea. Furthermore, the water status was not substantially affected, on the contrary to the carbon metabolism which was affected as reported in other studies (Chaves, 1991).

The parameters derived from analysis of the induction curve of maximum fluorescence of chlorophyll *a* are consistent with these ideas. A general decrease in *F*_V_/*F*_M_ and to a larger extend in PI was observed in response to the WD treatments, as expected due to the sensitivity of PSII to WD conditions (Maxwell and Johnson, 2000). PI is a global index of performance (expressed in analogy to the Nernst potential) which is composed of three components: the force due to the concentration of active reaction centers, the force of the light reactions which is related to the quantum yield of primary photochemistry and the force related to the dark reactions (Živčák et al., 2008). PI has been defined as a “drought factor index” by Goltsev et al. (2012) during desiccation of beans *Phaseolus vulgaris* L., which is in accordance with the present observations on tomato. Moreover, the increase in the quantum yield of the electron transport flux until PSI acceptors (*J*_0_^RE1^/*J*^ABS^) could be explained by a return of electrons from PSI to PSII named the cyclic electron flow (Johnson, 2011), which is described as an orchestrator of the chloroplast energy budget, that increases in response to environmental stressors such as high light, WD simulated by low CO_2_ supply, or extreme temperatures in higher plants (Livingston et al., 2010; Johnson, 2011; Walker et al., 2014). The significant increase in quinic acid which was observed in all accessions in response to the WD treatments could be related to the increase in *J*_0_^RE1^/*J*^ABS^. Indeed quinic acid has been described as a potential accelerator of the electron transport due to its capacity to act as a non-classical uncoupling factor on photophosphorylation (Barba-Behrens et al., 1993). Finally, the increase in chlorophyll content in some genotypes, which is not compatible with photodamage in leaves, contributes to the idea that there was an efficient acclimation to maintain photosynthetic activity under WD.

In summary, the shifts in energy fluxes around PSII, the accumulation of starch in leaves and the decrease in the specific leaf surface area, which is a recognized if not specific consequence of WD, are all potent indicators that plants submitted to WD were indeed stressed (Chaves, 1991; Zgallaï et al., 2005). They are also indicators of acclimation processes aiming at relieving the photosynthetic machinery from overheating, arguably as a consequence of decreased translocation to active growth areas. Finally, it appears that osmotic and photosynthetic regulations were highly involved in plant acclimation to successive episodes of WD. Such acclimation effects were observed in almost all accessions, but more clearly in Criollo, LA1420, and Stupicke. Overall, Cervil exhibited the weakest responses, suggesting that this genotype is poorly sensitive to WD as recently suggested by Albert et al. (2015).

### The WD Treatments Reduced Fruit Growth Proportionally to the Increase in Stress Intensity and to Cumulative Effects of WD

Depending on its intensity, WD is expected to decrease fruit size and fruit water content, thus increasing the metabolite contents through a concentration effect. WD may also stimulate the accumulation of osmotic and antioxidant compounds (Ripoll et al., 2014). Despite the absence of a significant response, fruit size and weight were mainly reduced in S2 fruits by the WD treatments (except in the cherry tomato Cervil). These observations are not in accordance with others studies, where WD was reported to have positive effects on fruit growth, due to a negative regulation of fruit setting and to an increase of carbon supply to the remaining fruits (Li et al., 1989; Vallverdu et al., 2012). In the present study, the plant fruit load was regulated at similar level in control and WD plants, thus the maintenance of fruit growth arguably resulted from osmotic regulations and/or sugar compartmentation in the fruit (Ripoll et al., 2015). The reduction of fruit size and weight in S2 fruits is consistent with the idea that fruit yield decreases proportionally to the intensity of WD (Wang and Gartung, 2010). Competition for carbon was likely higher during the development of S2 fruits compared to S1 fruits, due to the cumulative effects of the three WD periods on the plant carbon budget. Moreover fruit growth of large fruit genotypes was more impacted by the WD treatments than fruits of small fruit genotypes, arguably due to higher carbon demand for large fruit growth. Indeed, in WD conditions, sink organ growth was suggested to be reduced mainly through carbon dependent mechanisms (Muller et al., 2011). However, water fluxes are indirectly linked to carbon metabolism through osmotic and turgor regulations, as discussed below. So, the genotypic differences observed in response to the WD treatments were arguably driven by the additive effects of differences in water flux on fruit expansion, of source-related differences in carbon supply and of sink-related differences in carbon demand.

### The WD Treatments Maintain Fruit Sugar and Acid Contents

As for fruit fresh weight, the increase in DM content was higher in S2 fruits than in S1 fruits, suggesting that S2 fruits were submitted to higher stress intensity than S1 fruits. An important increase in S2 fruits DM content was observed in response to the WD treatments in the large-fruit genotype Levovil as well as in the cherry tomato type Cervil. On the contrary, changes in DM composition were more pronounced in S1 fruits than in S2 fruits and responses were highly dependent on genotypes. Variations in fruit composition in response to WD may result either from dilution/concentration effects (Guichard et al., 2001; Etienne et al., 2013), from active solute accumulation (Lo Bianco et al., 2000; Hummel et al., 2010), or from starch breakdown, as observed in tomatoes under salinity-induced WD (Balibrea et al., 2003). Soluble sugars and organic acids (primarily malic and citric acids) are major osmotic compounds that accumulate in fleshy fruits and determine fruit taste. Previous studies on tomatoes showed an increase in fruit sugar content under WD depending on cultivars and timing of stress (Veit-Köhler et al., 1999; Bertin et al., 2000; Chen et al., 2014). In the present study, the total content in soluble sugars on a DM basis was not strongly affected by the WD treatments except in Cervil fruits which also accumulated large amounts of starch and acids. Thus, in cherry tomato the accumulation of starch, soluble sugars, and acids may be an adaptive strategy to maintain the phloem-to-fruit gradient of sugars and regulate cell turgor, sustaining fruit growth in WD conditions. Sucrose content on a DM basis was the most affected by the WD treatments among soluble sugars, but it represents only a minor part (<3%) of total soluble sugars in these genotypes. On a fresh weight basis, the increase in fruit sugar content in response to WD was observed only in Cervil and Levovil, which questions the idea that WD has a positive impact on fruit taste (Ripoll et al., 2014) and suggests that such effect strongly depends on genotype and WD intensity. The effects of WD on fruit acidity are more conflicting (Etienne et al., 2013). In many species (peach, clementine *Citrus clementina* Hort ex. Tan, mandarin *Citrus reticulata* B., pear *Pyrus* L., tomato), water supply has been shown to negatively correlate with organic acid content in ripe fruits, but in grapes *Vitis vinifera* L., nectarines *Prunus persica var. nucipersica* L. (Etienne et al., 2013) and tomatoes (Mitchell et al., 1991; Veit-Köhler et al., 1999; Bertin et al., 2000), this correlation has been shown to be positive.

### Effects of the WD Treatments on Fruit Carotenoid and Ascorbic Acid Contents Ranged From Negative to Nil to Positive

Fruits supply a large range of health-promoting phytochemicals, of which secondary metabolites, primarily terpenoids (carotenoids, ABA, and others), and phenolic compounds, are the largest group along with AsA. Of all of the environmental factors that play a stimulating role in the synthesis and accumulation of useful phytochemicals in fruits, moderate stress, and more specifically, controlled drought may influence the metabolism of these phytochemicals via at least two major mechanisms that are not mutually exclusive and that may even interact (Nora et al., 2012; Poiroux-Gonord et al., 2013; Fanciullino et al., 2014). Firstly, drought typically induces a decrease in net photosynthesis which reduces the supply of primary metabolites to the fruits that are the major source of precursors for the biosynthesis of phenolic compounds, carotenoids, and AsA. Secondly, drought may exacerbate oxidative stress and signaling which is known to directly and indirectly influence the biosynthetic pathways of these compounds in leaves (Fanciullino et al., 2014). In the present study, the effects of the WD treatments on fruit carotenoid content ranged on a DM basis from negative, to nil to positive depending on genotype and stress intensity (S1 and S2 fruits). This is in complete agreement with divergent responses reported in the litterature (reviewed by Ripoll et al., 2014). Similarly, total AsA was reduced in S1 fruits of all genotype but one (LA1420), whereas more variable effects were observed in S2 fruits. Many studies reported positive effects of WD on AsA (Zushi and Matsuzoe, 1998; Veit-Köhler et al., 1999; Favati et al., 2009; Murshed et al., 2013), but also indicated variable effects depending on genetic and seasonal factors or the intensity and duration of the treatment. In S1 fruits, carotenoid accumulation (on a DM basis) was increased in four genotypes and reduced in four other genotypes including cherry tomato and large-fruit genotypes. Taken together, our observations confirm previous observations (Poiroux-Gonord et al., 2013) that tend to refute the hypothesis that the supply of carbon to fruit determines carotenoid synthesis. In tomato fruits, the absence of correlation between sugars and reduced AsA content also suggests that fruit AsA content is not limited by leaf photosynthesis or sugar availability (Gautier et al., 2009). Variations in carotenoids and AsA content would therefore result from stress-induced cellular redox changes (Fanciullino et al., 2014). In tomato plants, AsA content has been suggested to correlate with resistance to WD (Zhang et al., 2011; Garchery et al., 2013). However in the present study, only one genotype (LA1420) exhibited an adaptive response at the fruit level through an increase in both carotenoid and AsA contents. On a fresh matter basis, the fruit content in phytonutrients was improved by the WD treatments only in the cherry tomato Cervil and in the small fruit size genotypes (LA1420 and Plovdiv) through an increase in reduced AsA. This increase resulted mainly from concentration effects than from metabolic stimulation, in agreement with recent findings (Ripoll et al., 2015).

## Conclusion

In the present study, the WD treatments, which consisted in three successive cycles of moderate WD and recovery during the plant reproductive period, resulted in independent responses at the leaf and fruit levels. Considering parameters derived from chlorophyll *a* fluorescence measurements and leaf composition, we may hypothesize that for some genotypes the cyclic electron flow (extrapolated from *J*_0_^RE1^/*J*^ABS^) and quinic acid content were involved in energy dissipation and regulation of oxidative stress during the WD treatments. Negative effects on fruit fresh weight were dependent on stress intensity, while beneficial effects on fruit taste (sugars and acids) and nutritional value were weak or even negative. Interestingly, high starch accumulation in fruit could be a potential asset to sustain fruit growth under WD. Considering a large range of plant and fruit traits, our observations clearly show that responses to drought are highly variable and that they strongly depend on genotypic effects and on the stage of development at the time WD is applied. On their whole, the present results demonstrate that drought could be exploited positively, and that repeated cycles of WD and recovery may be used to improve fruit taste and at the same time minimize fruit size reduction. A strategy for breeding would be to stack in one single genotype adaptive traits at the leaf and fruit levels. To this end, small-fruit genotypes, in particular LA1420 and Criollo, represent an interesting potential source of traits of interest, as far as acclimation is concerned. However, our capacity to take full advantage of drought events or controlled WD is clearly conditioned by a shift in our way of thinking. We need to explore the full variability of genotypic responses by taking into account a much broader range of crop performance criteria than the ones that are usually considered and by systematically including observations made at different stages of development. The complexity revealed by our observations clearly suggests that exploring the variability of genotypic responses represents a difficult task, but then it is our belief that this is how the issue of drought on crop performance should be addressed from now on, and that the reward will come up to the challenge.

## Author Contributions

This work is part of the Ph.D. thesis of JR, who significantly contributed to the experiment, the biochemical analyses, the statistical analyses, and the redaction of the article. Original idea of this project was developed by NB and LU, who contributed to the experimental protocol, the redaction of the article, and to the mentoring and training of JR

## Conflict of Interest Statement

The authors declare that the research was conducted in the absence of any commercial or financial relationships that could be construed as a potential conflict of interest.

## Abbreviations

ANOVA: analysis of variance
AsA: ascorbic acid
AUC: area under the curve
DM: dry matter
MAGIC TOM: Multi-Parent Advanced Generation Inter-Cross population of Tomato
Plovdiv: genotype PlovdivXXIVa (*S. lycopersicum* L.)
PS: photosystems
RP: recovery period
RP1: recovery period 1
RP2: recovery period 2
RP3: recovery period 3
Stupicke: genotype Stupicke Polni Rane (*S. lycopersicum* L.)
WD: water deficit
WD1: water deficit period 1
WD2: water deficit period 2
WD3: water deficit period 3
Parameters: Ψ_predawn_: predawn water potential
Ψ_midday_: midday water potential
Ψ_soil_: soil water potential
*F*_V_*/F*_M_: maximum efficiency at which light absorbed by PSII is used for reduction of *Q*_A_
*J*_0_^RE1^/*J*^ABS^: quantum yield of the electron transport flux until PSI acceptors
PI: Performance Index (Strasser et al., 2004)
